# Indications for the addition of a patellofemoral joint arthroplasty following a previous unicondylar knee arthroplasty– a literature review and Delphi consensus

**DOI:** 10.1007/s00402-024-05738-z

**Published:** 2025-01-11

**Authors:** Gareth G. Jones, Stefano Campi, Fabian von Knoch, Alexandre Lunebourg, Nick London, David Barrett, Jean-Noel Argenson

**Affiliations:** 1 MSk Lab, Imperial College London, London, UK; 2 Campus Bio Medico University Hospital, Rome, Italy; 3 Fondazione Policlinico Universitario Campus Bio-Medico, Rome, Italy; 4 Zurich Bone and Joint Center, Zurich, Switzerland; 5 CCAL, Yverdon Hospital, Yverdon-les-Bains, Switzerland; 6 Yorkshire Knee Clinic, Leeds, UK; 7 Leeds Beckett University, Leeds, UK; 8 King Edward VII Hospital, Windsor, UK; 9 School of Engineering Science, University of Southampton, Southampton, UK; 10 Institute for Locomotion, Aix-Marseille University, Marseille, France; 11 CNRS 5, St. Marguerite Hospital, Marseille, France

**Keywords:** Unicompartmental knee arthroplasty, Patellofemoral joint arthroplasty, Bicompartmental knee arthroplasty, Revision knee arthroplasty, Compartmental knee arthroplasty

## Abstract

**Introduction:**

The aim of this study was to establish an international consensus statement on the indications for the addition of a patellofemoral joint arthroplasty (PFJA) in patients with a unicondylar knee arthroplasty (UKA) and symptomatic progression of patellofemoral compartment osteoarthritis.

**Materials and methods:**

A systematic review of the literature was conducted, and the results used to inform the development of a statement by an expert working group. This was then evaluated and modified, using a Delphi process, by members of the European Knee Society (EKS).

**Results:**

Forty-nine (round one) and forty-two (round two) EKS members took part in the Delphi process, with 83% agreement on the resulting consensus statement that the indications for this procedure are: (1) a well-functioning UKA in a satisfied patient with secondary osteoarthritis progression in the patellofemoral compartment (2), symptomatic patellofemoral compartment osteoarthritis with full thickness cartilage loss affecting the lateral facet of the patellofemoral joint (3), functional ligaments, including the anterior cruciate ligament (ACL) (4), a lateral tibiofemoral compartment with no cartilage damage greater than Ahlback Grade 1 (5), knee flexion ≥ 100° and extension loss ≤ 5° and (6) older patients with increased medical co-morbidities.

**Conclusions:**

The simple addition of a PFJA to patients with an existing UKA and progression of patellofemoral compartment osteoarthritis is an attractive option. This EKS Delphi-derived consensus statement, which reached a strong consensus, can be used by clinicians to identify patients suitable for this procedure.

## Introduction

In appropriate candidates, unicondylar knee arthroplasty (UKA) affords a number of benefits to both the patient and healthcare system [1]. Revision rates for UKA are comparable to total knee replacement (TKA) when performed by higher volume surgeons [2], but some patients will of course require further surgery during their lifetime. For UKA, progression of osetoarthritis is the most common cause of reoperation, accounting for approximately 30% of cases [3]. This can involve either the patellofemoral compartment, the remaining native tibiofemoral compartment, or both. In patients with isolated symptomatic progressive patellofemoral osteoarthritis, the surgical treatment is a choice between the addition of a patellofemoral joint arthroplasty (PFJA), or revision to a TKA.

In general, UKA to TKA is considered a relatively simple revision procedure, and in most cases can be achieved with a primary total knee arthroplasty prosthesis [4]. However, sometimes revision components, such as stems and wedges, are required to address bone loss following removal of a well fixed UKA [5]. The alternative option of adding a PFJA to a knee with an existing UKA has theoretical advantages, being a smaller and more bone conserving procedure which maintains the cruciate ligaments, and in doing so may improve knee function and satisfaction through improved proprioception and kinematics [6, 7]. In silico it has also been shown to result in a more natural load transfer to the underlying bone, which may help preserve bone stock and prevent the problem of periprosthetic fractures seen with total knee replacements [8, 9].

Despite the potential advantages of the compartmental approach to revision knee surgery, there remains a lack of evidence-based guidelines to assist surgeons in identfying suitable patients. The purpose of the current study was to develop an international consensus statement on the indications for the addition of a PFJA in patients with a UKA and progressive patellofemoral compartment osteoarthritis.

## Materials and methods

### Literature review

Medline and Embase databases were searched from 1947 to October 18th 2022. The following three keywords (and their related synonyms) were used to develop a sensitive search strategy: “patellofemoral joint arthroplasty”, “unicompartmental knee arthroplasty” and “bicompartmental knee arthroplasty”. All synonyms relating to the these words were combined by applying the Boolean command “OR”. Truncated search terms utilising the wildcard character, and the “ADJ2” operator, were used to broaden the search. References of included articles were also manually searched to identify any other studies of interest.

Two investigators (GGJ and SC) manually screened the titles and abstracts for inclusion, prior to reviewing the full-texts. Studies were considered eligible if they met the defined PICO criteria: patients with a partial knee replacement who develop symptomatic patellofemoral osteoarthritis (population) and undergo a patellofemoral joint replacment (intervention), compared to either no surgical intervention, or a total knee replacement (comparison). The outcome measures of interest were revision rate, functional outcomes, morbidity and mortality. Non-human studies, review articles, editorials, case reports, letters, conference abstracts, unpublished studies, and studies not written in English were excluded. Studies pertaining to monolithic designs of patellofemoral joint replacements were also excluded given that these have been withdrawn from the market.

### Delphi consensus

The European Knee Society (EKS) Delphi process uses the methodology described by Cats-Baril et al. [10] together with its subsequent modifications [11]. Seven EKS surgeons constituted a working group, who synthesised the results of the literature review to develop a statement on the indications for PFJA following a UKA. Members of EKS who attended the society’s meetings, either in-person or remotely, participated in the Delphi process. The definition of consensus was based on the description published by Linstone et al. [12].

During the first round, EKS members were asked to ‘agree’ or ‘disagree’ with the following statement:

*The addition of a patellofemoral joint arthroplasty (PFJA) in patients with a unicondylar knee arthroplasty (UKA) and progression of patellofemoral osteoarthritis is a reasonable alternative to a total knee arthroplasty (TKA) if the following criteria are fulfilled*:

1. *Confirmation that the pain is secondary to patellofemoral joint osteoarthritis*.
2. *A well-functioning UKA with no evidence of malalignment*,* wear or loosening*.
3. *The remaining native tibiofemoral compartment must be intact*.
4. *Functional ligament stability*.
5. *Younger age*,* or medically high risk for revision surgery*.

Informed by the voting result and discussion during the first round, revisions were made to the statement for the second round:

*The indications for the addition of a patellofemoral joint arthroplasty (PFJA) in patients with a unicondylar knee arthroplasty (UKA) and progression of patellofemoral osteoarthritis are*:

1. *A well-functioning UKA in a satisfied patient with secondary osteoarthritis progression in the patellofemoral compartment*.
2. *Symptomatic patellofemoral compartment osteoarthritis with full thickness cartilage loss affecting the lateral facet of the patellofemoral joint*.
3. *Functional ligaments*,* including the anterior cruciate ligament (ACL)*.
4. *A lateral tibiofemoral compartment with no cartilage damage greater than Ahlback Grade 1*.
5. *Knee flexion ≥ 100° and extension loss ≤ 5°*.
6. *Older patients with increased medical co-morbidities*.

## Results

### Literature review

**Fig. 1:**
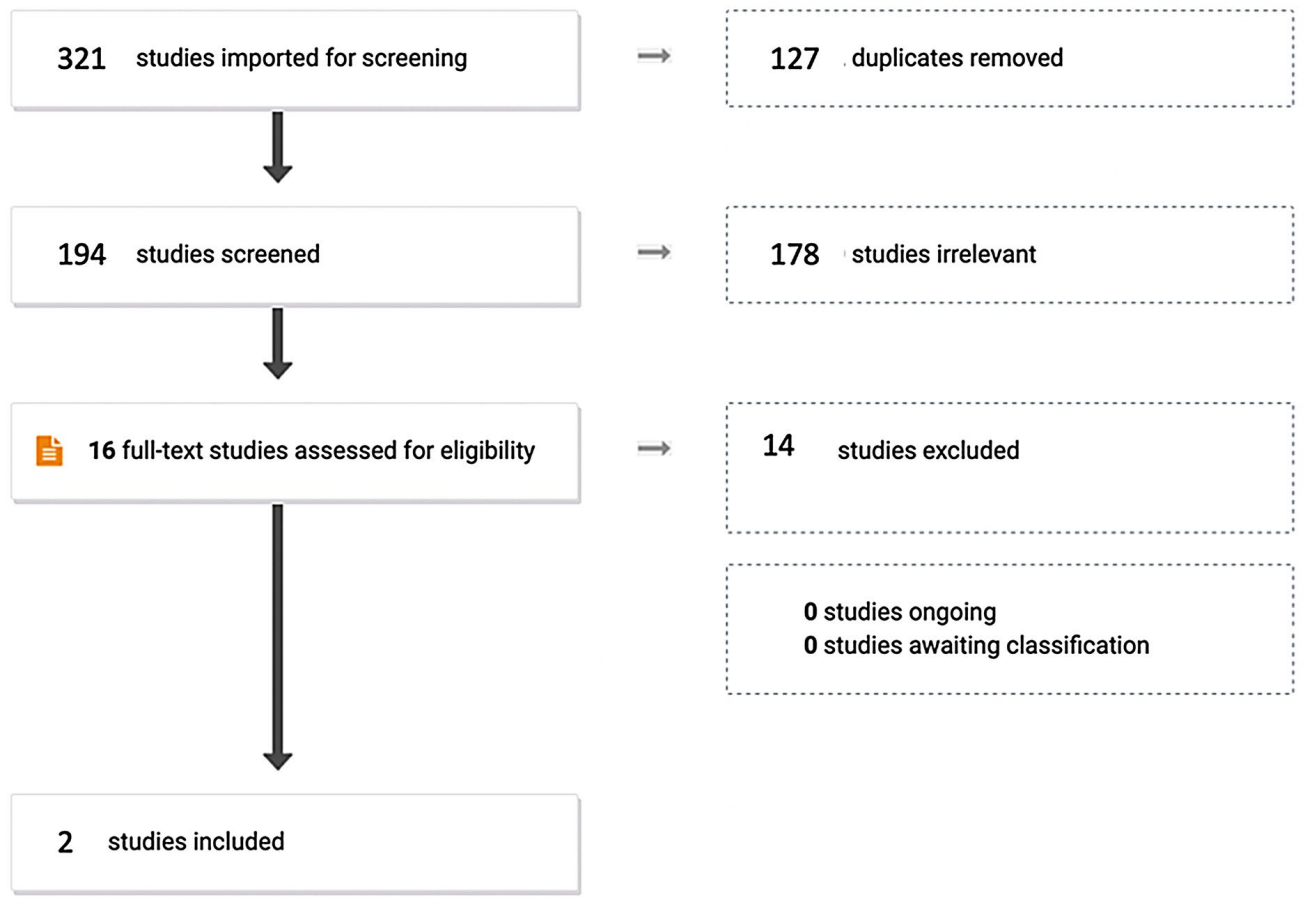
PRISMA flow diagram

Two studies, both comparative in nature, were considered eligible for inclusion in the literature review (see *PRISMA* diagram in Fig. 1).

Haffar et al. [13] reported on a retrospective case series of 27 patients who underwent staged compartmental surgery for progressive osteoarthritis, and compared their outcomes to a group of 30 patients who underwent revision to a total knee replacement for the same indication. Indications for a compartmental approach were: ‘knee stability, an intact ACL, and arthritis progression in one other compartment’. They report equivalent survivorship to TKA, but only three patients in the compartmental group received a PFJA, and their outcomes were not considered separately.

Garner et al. [14] retrospectively studied a group of 23 patients with progressive osteoarthritis who were treated with a compartmental approach, objectively comparing their gait with 23 matched primary total knee replacement patients, and 22 healthy subjects. Overall, the compartmental group had more normal gait characteristics, but only six out of the patients in this group underwent addition of a PFJA, and their results were not reported separately. Indications used for inclusion in the compartmental group were: osteoarthritic degeneration of a single native compartment, well-functioning primary partial knee arthroplasty in situ, functional anterior cruciate ligament (relative contraindication in the elderly provided the knee is otherwise stable), correctable varus/valgus and medically high risk for revision surgery. Contra-indications for inclusion in the compartmental group were: osteoarthritic degeneration of two native compartments, loose/unstable/problematic primary partial knee arthroplasty, anterior cruciate ligament dysfunction, inflammatory arthropathy, or evidence of periprosthetic infection.

### Delphi

Forty-nine active EKS members participated in round one of the Delphi: 39 (80%) agreed with the statement provided by the working group, 5 (10%) disagreed, and 5 (10%) abstained. Forty-two active EKS members participated in round two of the Delphi: 35 (83%) agreed with the statement, 4 (10%) disagreed and 3 (7%) abstained

## Discussion

This study highlights the paucity of evidence in the literature regarding patient outcomes following the addition of a PFJA in patients with an existing UKA and progressive patellofemoral compartment osteoarthritis. Only two published studies of patients undergoing this procedure were identified, with small numbers, and results only reported as part of a larger heterogenous group undergoing compartmental revisions. This means that any inference regarding outcomes for this procedure is impossible, and increases the potential utility of the expert consensus to guide clinical practice

After two rounds of the Delphi, the statement on the indications for the addition of a PFJA in patients with a UKA and progressive patellofemoral compartment osteoarthritis reached a super majority/strong consensus [12], which means that it is appropriate for clincial use. Understandably, the statement includes the proviso that the UKA must be well functioning and that the diagnosis of secondary patellofemoral joint osteoarthrosis is confirmed prior to considering adding a PFJA [13–15]. In addition, given that adding a patellofemoral arthroplasty is effectively a staged medial bicompartmental knee replacement, it was recommended that the criteria agreed in a separate Delphi consensus regarding the indications for a primary bicompartmental arthroplasty should also be applied in its entirety to this procedure [16]. Addition of a PFJA instead of revision to a TKA was particularly recommended in older patients with associated medical issues, given that it is less invasive

This study does have limitations. The lack of published evidence to support the development of the consensus statement is recognised. However, the Delphi technique was robust, and is designed to address this scenario. Additionally, all seven members of the working group, and eighty-one experts who took part in the Delphi are members of the European Knee Society (EKS), whose strict entry requirements ensure an appropriate level of expertise. Whilst there are some international EKS members, the consensus statement may not be generalisable to parts of the world where practices and patients may differ

## Conclusion

Whilst the majority of UKAs can be simply revised to a primary total knee arthroplasty, some patients require more extensive surgery due to bone loss during implant removal, resulting in increased levels of fixation and constraint, which can adversely impact outcomes [17]. This makes the simple addition of a patellofemoral joint replacement in this cohort an attractive option. There is a clear need for large, well-constructed randomised controlled studies comparing this treatment option to revision TKA, but in the meantime, the expert Delphi-derived consensus statement developed in this study, which reached a strong consensus, can be used by clinicians to identify potential candidates for this procedure

## Data Availability

No datasets were generated or analysed during the current study.
